# Case report of patient with a Cronkhite-Canada syndrome: sustained remission after treatment with corticosteroids and mesalazine

**DOI:** 10.1186/s12876-019-0944-x

**Published:** 2019-02-27

**Authors:** Sigrid Schulte, Fabian Kütting, Jessica Mertens, Thomas Kaufmann, Uta Drebber, Dirk Nierhoff, Ulrich Töx, Hans-Michael Steffen

**Affiliations:** 1 Department of Gastroenterology and Hepatology, University Hospital of Cologne, D-50937 Cologne, Germany; 2 Marienhospital, Mühlenstrasse 21, D-50321 Brühl, Germany; 3 Institute of Pathology, University Hospital of Cologne, D-50937 Cologne, Germany

**Keywords:** Cronkhite-Canada syndrome, Mesalazine, Non-hereditary polyposis, Alopecia, Onychodystrophy

## Abstract

**Background:**

Cronkhite-Canada syndrome is a rare disease of unknown etiology and the optimal treatment for this syndrome is unknown.

**Case presentation:**

We present the case of a man who at the age of 66.0 years was diagnosed with Cronkhite-Canada syndrome (CCS). In addition to watery diarrhea, alopecia, and a complete loss of toenails and fingernails, the patient had been suffering from dysgeusia and rapid weight loss of more than 10.0 kg within a few months. The patient had recently incurred a distal radius fracture. During the initial endoscopy an extensive polyposis of the stomach and jejunum was found. The diagnosis of CCS was made and after initiation of a steroid therapy his diarrhea improved immediately. A discontinuation of the steroid therapy was not possible and mesalazine (1000 mg t.i.d.) was added to prednisolone (10.0 mg/d). This therapy led to a remission within 6.0 months with weight gain and normalization of serum albumin levels. The prednisolone dose was reduced to 7.5 mg/d. During the following year, the steroids could be further reduced and nails had regrown again. Within three years, all polyps had disappeared and the steroid therapy was finished while the dosage of mesalazine was reduced in a stepwise fashion. Four years later, the mesalazine was stopped and more than 14.0 years after the initial diagnosis the patient is still in complete remission without any treatment.

**Conclusion:**

The optimal treatment for CCS is unknown. In our case, the initial combination therapy of corticosteroids plus mesalazine followed by a mesalazine monotherapy has led to a remarkable long-lasting remission with complete resolution of all intestinal polyps.

## Introduction

Cronkhite-Canada syndrome is a rare non-hereditary disease of unknown etiology, first described in 1955 [1, 2]. Alongside a gastrointestinal hamartomatous polyposis and gastrointestinal symptoms such as diarrhea, other symptoms such as onychodystrophy, alopecia, hyperpigmentation of the skin, and rarely vitiligo [2] are associated with this condition. Its pathogenesis remains unclear, however, an autoimmune process is suspected due to increased IgG4 levels found in CCS polyps [3]. More than 500 cases have been described in the literature, 75.0% originate from Japan. The average age of onset is 59.0 years with a 3:2 male/female distribution [4].

### Clinical course

Diarrhea represents the leading symptom in 80.0% of cases and as the initial symptom in 35.0% of all cases. Further symptoms include dysgeusia (41.0%), dry mouth (6.0%), and upper abdominal pain (9.0%). The disease tends to follow a progressive course with a high mortality as a result of cachexia, anemia, sepsis and shock [5, 6]. The 5-year-mortality rate is 55.0%, primarily due to gastrointestinal bleeding, infections, malnourishment, and severe electrolyte imbalance [4, 5, 7]. Besides fulminant and chronic disabling cases complete remissions have been described; however, these occur in only 5.0% of all cases [8]. Gastrointestinal polyposis due to CCS is principally non-neoplastic and rather inflammatory/hamartomatous in nature. Cases of malignant polyps following a typical adenoma-carcinoma sequence have been described [9–14] as well as colorectal carcinoma after malignant transformation of serrated polyps [15]. The elevated risk for colorectal carcinoma calls for aggressive screening in these patients. However, the detection of adenoma among the great number of hyperplastic inflammatory polyps is challenging [16]. Ward and Wolfsen recommend systematically resecting all polyps > 1.0 cm in order to prevent the development of colorectal carcinoma [8, 17].

### Differential diagnoses

Ménétrier disease and other polyposis syndromes such as familiar adenomatous polyposis, Gardner syndrome, juvenile polyposis, Peutz-Jeghers syndrome and Turcot syndrome have to be taken into consideration. Furthermore, protein-losing enteropathies such as chronic inflammatory bowel diseases, Whipple’s disease, or intestinal lymphangiectasia have to be ruled out [8, 18].

## Case presentation

**Fig. 1:**
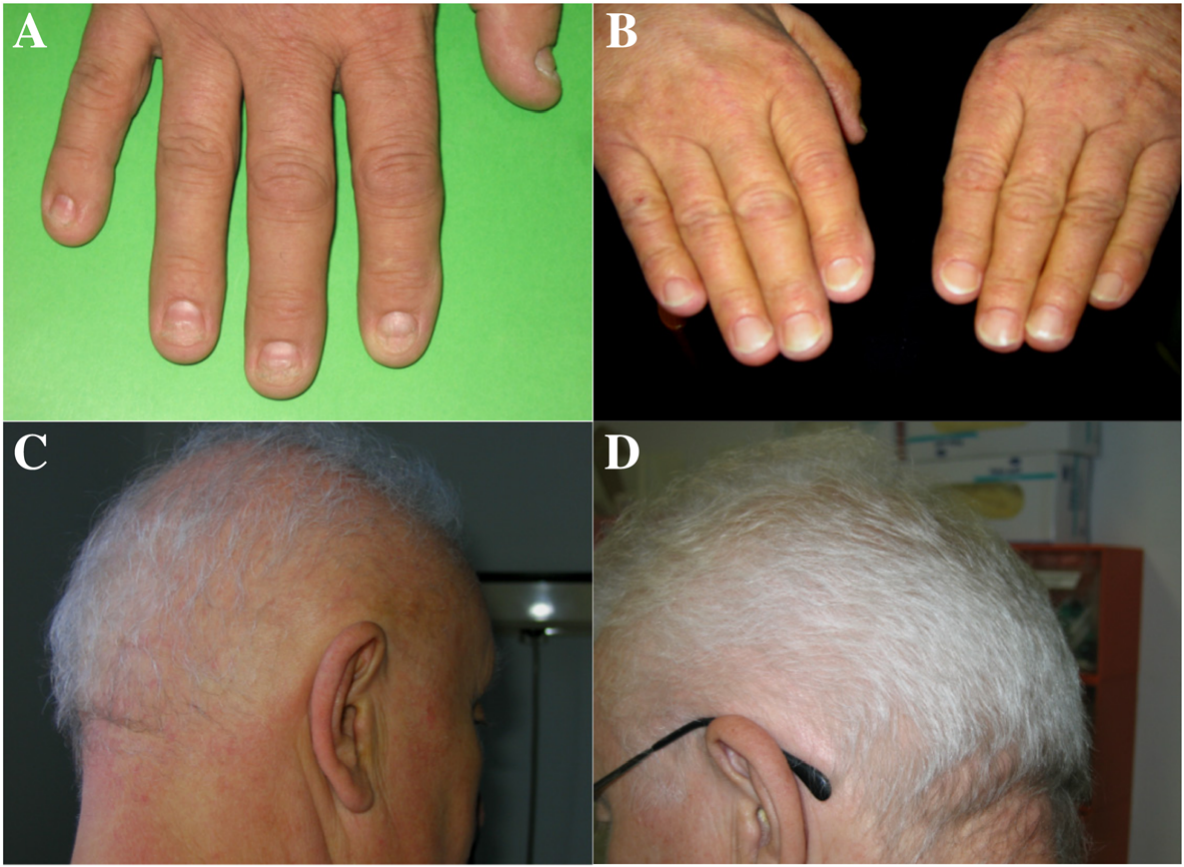
Ectodermal alterations before (A/C) and after (B/D) combined therapy with corticosteroids and mesalazine. **a** Finger nail dystrophy, **b** regular nails, **c** alopecia, **d** regular hair growth

Upon first presentation to his local hospital, our patient was 66.0 years old and initially complained of diarrhea, alopecia and dystrophy of his nails (Fig. 1 A, C). He also had recently incurred a distal radius fracture. He described dysgeusia and had a reported weight loss of 10.0 kg in the past few months. He was a non-smoker and did not drink alcohol. His family history revealed no cases of gastrointestinal polyposis or colorectal malignancies.

The diagnosis of Cronkhite-Canada syndrome (CCS) was quickly established and the patient was treated with corticosteroids (prednisolone 50 mg orally q. d.). This led to a prompt response, especially his diarrhea improved markedly within a short period of time.

Since his radial fracture did not develop a proper callus, tapering of steroids had been tried, however, relapsing severe diarrhea required the prompt step-up in dosage.

Eleven months after the initial diagnosis the patient was transferred to our hospital due to ongoing malnourishment (Table 1). Laboratory workup showed severe iron deficiency with microcytic anemia, hypoproteinemia, and hypalbuminemia. The patient’s blood pressure was low at 110/70 mmHg.

**Table 1:** Laboratory results on admission to our clinic (9 months after initiation of steroid therapy) and at the end of treatment

Parameter	On admission	At the end of therapy	Normal range
Hemoglobin (g/dl)	9.2	13.3	13.5–18
MCV (fl)	72	86	80–96
Leucocytes (x1E9/l)	7.48	6.90	4.4–11.3
Thrombocytes (x1E9/l)	369	171	150–400
Erythrocytes (x1E12/l)	4.3	4.5	4.4–5.9
Potassium (mmol/l)	3.0	4.3	3.6–4.8
Chloride (mmol/l)	107	103	94–110
Magnesium (mmol/l)	0.67	0.73	0.7–1.1
Calcium (mmol/l)	2.29	2.45	2.20–2.65
Total protein (g/l)	60	75	66–83
Albumin (g/l)	34	45	35–52
ALT (U/l)	16	12	10–50
AST (U/l)	13	17	10–50
LDH (U/l)	178	165	135–225
Iron (μmol/l)	2.6	20.4	9.0–30.0
Ferritin (μg/l)	4	166	30–400
Transferrin (g/l)	2.83	2.90	2.00–4.00
Transferrin saturation (%)	4	30	16–45
Amylase (U/L)	29	30	13–53
Lipase (U/l)	32	35	13–60
CRP (mg/l)	< 3	< 3	< 8
ESR (mm/h)	29	16	< 14
TSH (mU/l)	0.84	2.80	0.27–4.20
Zinc (μmol/l)	8.7	11.7	10.6–17.9
Vitamin B12 (ng/l)	214	282	181–701

**Fig. 2:**
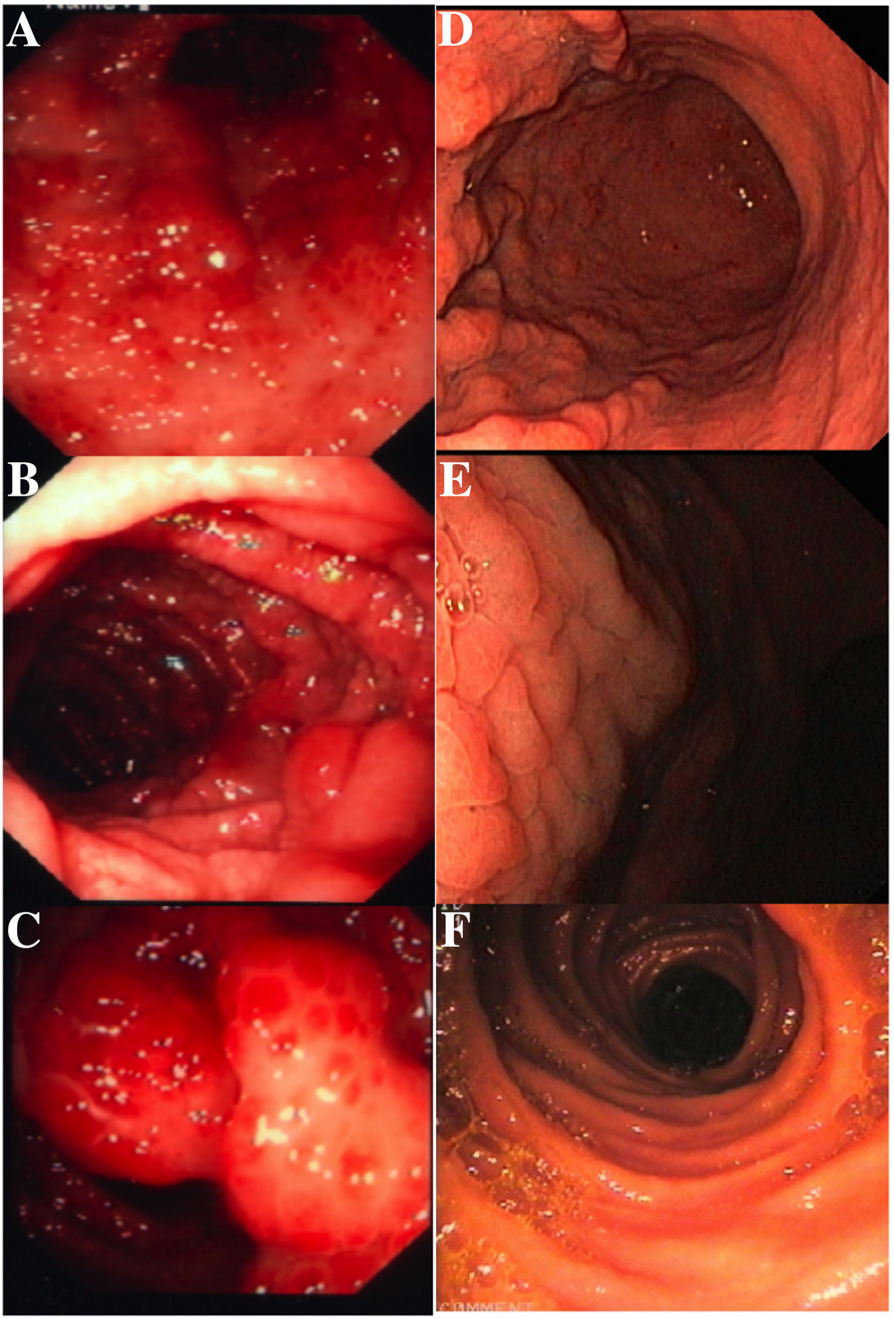
Severe polyposis before initiation of combined therapy with corticosteroids and mesalazine. (**a**: stomach; **b**, **c**: colon); complete remission 3 years after conversion to monotherapy with mesalazine (**d**: stomach, **e**, **f**: colon)

**Fig. 3:**
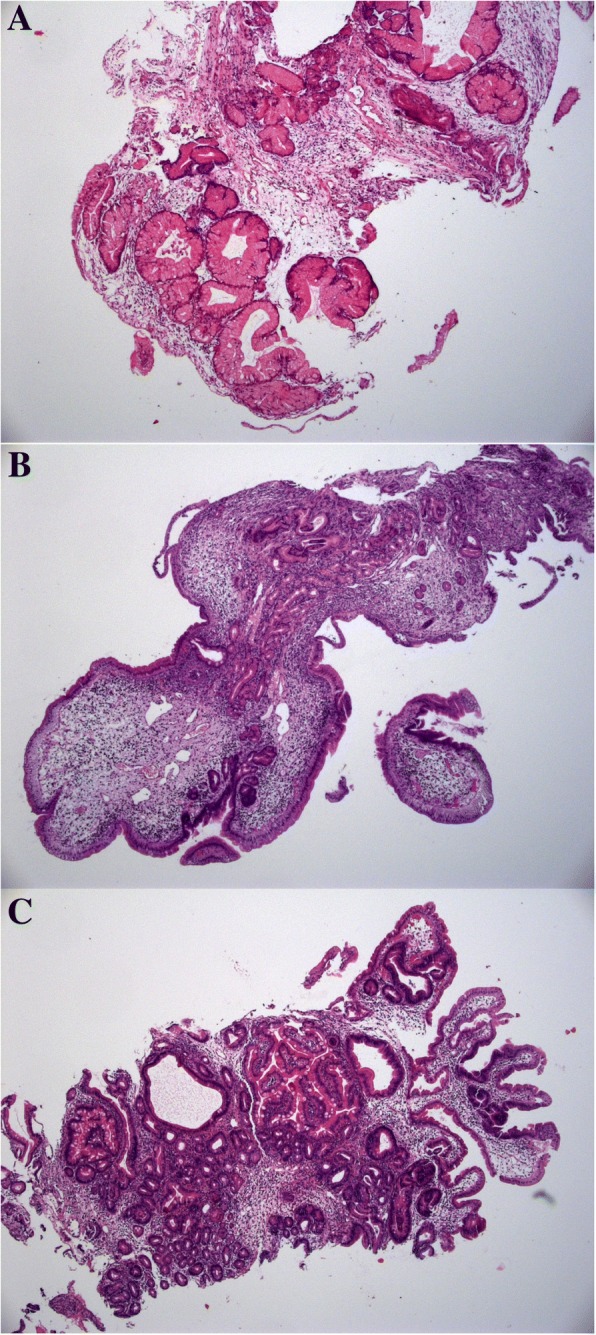
Histology: gastric mucosa with cystic enlargement of crypts, edema and inflammatory stroma infiltrates, mainly consisting of plasma cells and lymphocytes (**a**). Duodenal polyp with edema und inflammatory stroma infiltration (**b**). Jejunal polyp with cystic elongation of glands, edema and inflammatory stroma infiltration (**c**)

Barium contrast enema showed multiple polypoid lesions throughout the colon as well as smaller lesions in the upper GI tract. Endoscopies of the stomach, upper and lower GI tract confirmed these findings, showing an extensive polyposis of the stomach, the small intestine as well as the colon. The size and numbers of the broad based polyps decreased aborally and their endoscopic appearance resembled the typical aspect of polyps upon polyps (Fig. 2). The gastric tissue biopsies showed cystic enlargement of crypts with edema and inflammatory stroma infiltrates, mainly consisting of plasma cells and lymphocytes. The analysis of duodenal and jejunal polyps resulted in similar findings with cystic enlargement of mucine containing glands, a stromal edema and a dense inflammatory infiltrate (Fig. 3). IgG4-immunohistochemistry showed a very faintly positive signal.

After ruling out malabsorption, bacterial overgrowth, lactose intolerance or celiac sprue, the patient was put on a diet rich in protein as well as zinc supplements, and mesalazine (1000 mg orally t.i.d.) was administered together with prednisolone at a dose of 10.0 mg/d.

A combination of prednisolone and mesalazine 1500 mg/d had been proven to be effective with regard to symptoms and regression of colonic polyps in an earlier published case report [19]. However, we decided to use a higher mesalazine dose well within the recommended range (2.0–4.8 g/d in three divided doses) for the oral treatment of ulcerative colitis at that time [20].

This therapy led to a weight gain of 9 kg during the following 6 months and a normalization of serum albumin levels, while serum zinc levels remained low. Hence, the prednisolone dose was reduced to 7.5 mg/d, the mesalazine dose remained the same. During the following year, steroids were further tapered and the patient’s nails regrew (Fig. 1b and d) and a repeated endoscopy of the stomach and proximal small intestine showed a reduced number of polyps. At this point it was decided to stop the steroids and three years after the initiation of mesalazine therapy, all polyps had disappeared. The dosage of mesalazine was kept at 1000 mg t.i.d. for another year, followed by 500 mg t.i.d. for one year with a stepwise reduction (500 mg each year) thereafter. Finally, mesalazine was stopped and more than 14 years after the initial diagnosis the patient is still in complete remission without any treatment.

## Discussion and conclusions

Optimal medical therapy in CCS remains unclear and several strategies have been described. Corticosteroids improve exudative enteropathy in 90.0–93.0% of all patients [4]. Targeted diets and supplements are used to treat malnutrition and ectodermal alterations [1, 17, 19, 21, 22]. Antibiotics are used to combat bacterial overgrowth or to treat co-infections and septicemia [17]. Corticosteroids are recommended as the mainstay of medical treatment and have been used in various combinations, e.g. antiplasmin therapy, H2-recepter antagonists, proton pump inhibitors, or cromolyn sodium. Takakura et al. [19] were the first to describe a direct response to a combination therapy of prednisolone (25 mg/d) together with mesalazine (1500 g/d) leading to a reduction in polyposis, abdominal pain and diarrhea within 6 months. This regimen also had positive effects on upper GI polyposis [23].

Mesalazine is known for its role in the treatment of chronic inflammatory bowel disease, it reduces pro-inflammatory prostaglandins and leukotrienes and inhibits cytokines. Independent from its anti-inflammatory features it appears to have an antiproliferative effect on colorectal adenomas [24].

The exact mechanism of action, especially in the upper GI tract, including in our case, remains unclear. Our patient received Pentasa® (Ferring Pharmaceuticals, Copenhagen, Denmark) consisting of ethylcellulose-covered microgranula which continuously release their content from duodenum to ileum in a pH- and time-dependent way allowing systemic absorption (25.0 to 30.0%) during the passage of the small intestine [25–27].

In our case, the patient showed a good initial response to corticosteroid therapy, paired with nutritional supplements. However, we were faced with the problem of continued malnutrition and a non-healing distal radius fracture which required tapering steroids in the long run. This resulted in our decision to initiate mesalazine therapy and we opted for a dose within the recommended range (2.0–4.8 g/d in three divided doses) for the treatment of ulcerative colitis at that time [20].

Recently another case of CCS with longstanding remission has been published, however, in that case anti-inflammatory and immunosuppressive medications in addition to nutritional support resulted in sustained disappearance of clinical manifestations as well as the persistence of physical and psychological well-being over more than eight years [28]]. Our case also argues against the poor prognosis previously ascribed to patients with this syndrome. The successful combination of mesalazine with prednisolone in CCS as initially published by Takakura et al. [19] was confirmed and additionally a longstanding remission following a four year mesalazine monotherapy was shown.

## Abbreviation

CCS: Cronkhite-Canada syndrome
